# Personal

**Published:** 1900-01

**Authors:** 


### PERSONAL.

DR. LEONARD WOOD, Major-General U. S. Volunteers, formerly military Governor of Santiago de Cuba, has been assigned to the command of the division of Cuba, relieving Major-General John R. Brooke, U. S. Army.

Maj.-Gen. Wood will, in addition to his duties as division commander, exercise the authority of military governor of the island.

Gen. Wood on his arrival at Havana, December 20, 1899, was given a warm reception by officials and inhabitants. Salutes were fired, bunting displayed, rockets sent up, bands played and official visits exchanged. A new era for Cuba appears to be dawning under the administration of the new Governor-General.

---

Dr. F. W. McGuire, of Buffalo, has moved his office from 158 Northland Avenue, to 1540 Main Street, over Kingston's drug store. Hours: 8 to 9 a. m., 2 to 3, and 7 to 8 p. m. Residence, 158 Northland Avenue. Telephone.

---

Dr. Frederick H. Mills, of Buffalo, having completed his duties as acting assistant surgeon, United States Army, announces his return to practise, with office at No. 724 Main Street.

---

Dr. F. P. Bingham, of Buffalo, who has for some time been at the Fitch Hospital as assistant house surgeon, has become a member of the staff of the German Deaconess's Home.

---

Dr. Earl P. Lothrop, of Buffalo, who has been seriously ill from blood poisoning has so far recovered as to give promise of his early return to practice.

